# Critical Care Registries: The Next Big Stride?

**DOI:** 10.5005/jp-journals-10071-23227

**Published:** 2019-08

**Authors:** Bharath Kumar Tirupakuzhi Vijayaraghavan, Ramesh Venkatraman, Nagarajan Ramakrishnan

**Affiliations:** 1-3 Department of Critical Care Medicine, Apollo Hospitals, Chennai, Tamil Nadu, India

## Abstract

**How to cite this article:** Vijayaraghavan BKT, Venkatraman R, Ramakrishnan N. Critical Care Registries: The Next Big Stride? Indian J Crit Care Med 2019;23(8):387.

Dear Sir,

We read with interest the publication by Manu Varma and colleagues.^1^ We congratulate the authors for the admirable effort in capturing trends over a 12-year period from a large intensive care unit (ICU) in India. The study provides rich information on patient demographics, illness severity and outcomes, and as the authors acknowledge, the secular trends are in keeping with expected evolutions in practice patterns.

The study also demonstrates the feasibility of an electronic database (CHITRA) in collating data from ICUs and in monitoring case-mix patterns and outcomes. As critical care training programs expand and with the establishment of newer public and private ICUs across the country, there is a need for regional and national electronic registries to monitor case-mix patterns, quality indicators and outcomes. Several high income countries (HICs) have have well established registries such as UK (ICNARC-https://www.icnarc.org/), Australia/New Zealand (ANZICS CORE-https://www.anzics.com.au/anzics-registries/) and Sweden (SIR-https://www.icuregswe.org/en/).

In contrast to these HICs, the challenges for the successful establishment of country-wide registry in India and other middle income and lower-middle income countries (LMICs) are unique and manifold. The barriers include, but are not limited to, availability of data collectors, financial support models for data collection, lack of resources such as computers and a reliable basic internet plan, the reluctance of hospital administrators to share data, genuine patient privacy concerns and lack of awareness on the importance and value of consolidated data from large databases among clinicians and healthcare providers. Brazil is one of the middle-income countries that has successfully developed and implemented a large ongoing ICU registry.^2^ In the Indian sub-continent, the Network for Improving Critical Illness Training (NICST) (https://nicst.com/) has collaboratively developed registries in Sri Lanka (a national network of approximately 100 linked ICUs) and in Pakistan (> 20 linked ICUs).^3,4^ In Southern India, our group over the past 6 months has collaboratively developed a regional critical care registry- Indian Registry of IntenSive care (IRIS) which has successfully managed to bring 10 ICUs on a common cloud-based platform.

A key challenge to data collection is the ability to strike a balance between granularity of data and managing data collection burden. More variables and more details provide deeper insights but comes at the cost of data collector fatigue, missing data and sometimes a lack of direction and focus.^5^ In the absence of universal Electronic Health Record (EHRs) where seamless data flow is possible, this represents a substantial challenge. This problem is not unique to India or other LMICs but is certainly amplified in resource limited settings. IRIS has been adapted on a platform common to the Sri Lankan and Pakistan registries and the key strength of the model has been the ease of data collection. We have consciously chosen to keep mandatory data collection minimal, while at the same time, offering motivated units the opportunity to capture more information. This, we hope, will ensure that key information is available, yet minimize data collection burden and information loss. We look forward to sharing our experiences and learnings at future scientific meetings.

We believe there is an urgent need for an investment by professional societies in establishing both disease-specific (e.g. sepsis) and general registries to ensure that quality standards are adhered to and to offer opportunities for understanding secular trends in case-mix and outcomes. In a large country like India with wide variations in the delivery of intensive care and the inherent tension between public and private healthcare systems, the admirable goal of a single nation-wide registry may be impractical in the near term. Encouraging the development of regional registries to start with may be an attractive solution. IRIS represents one such effort. If well-coordinated and implemented, regional cloud-based registries may also be able to “talk to each other”, thus enabling greater synchrony and collaboration. Additionally, from the perspective of critical care societies, this will also crucially offer a launchpad for registry based clinical trials and for big data analytics. Registries can thus serve the triple purpose of quality monitoring, quality improvement and research.

We once again commend the authors of the stride study for an excellent effort and for taking a lead in showing us the way.
